# Influence of the acuity of patients’ illness on effectiveness of early, goal-directed mobilization in the intensive care unit: a post hoc analysis

**DOI:** 10.1186/s13054-020-03346-y

**Published:** 2020-11-25

**Authors:** Ludwig Scheffenbichler, Bijan Teja, Flora Scheffenbichler, Manfred Blobner, Timothy Houle, Matthias Eikermann, SOMT Team, Timothy Houle, Stefan Schaller, Karen Waak, Nicole Mazwi, Maximilian Hammer, Stephanie Grabitz, Karuna Wongtangman, Omid Azimaraghi

**Affiliations:** 1 Department of Anesthesia, Critical Care and Pain Medicine, Beth Israel Deaconess Medical Center, 330 Brookline Ave, Boston, MA 02215 USA; 2 Department of Anaesthesiology and Intensive Care Medicine, Technical University of Munich, Munich, Germany; 3 Department of Anaesthesia, Massachusetts General Hospital, 55 Fruit St, Boston, MA 02114 USA; 4 Department of Anesthesia, Critical Care and Pain Medicine, Beth Israel Deaconess Medical Center, Harvard Medical School, 375 Longwood Ave, Boston, MA 02215 USA; 5 Departments of Anesthesia and Critical Care Medicine, St. Michael’s Hospital, Toronto, ON Canada; 6 Department of Anesthesiology and Critical Care, University Hospital Ulm, Ulm, Germany; 7 Clinic for Anesthesiology and Intensive Care, Essen University Hospital, Essen, Germany

Dear Editor,

Early, goal-directed mobilization does not consistently translate into long-term functional benefits [1], which might be explained by inflammation and catabolism in high acuity patients, among other factors [2]. On the opposite end of the acuity spectrum, patients with low acuity may have favorable functional recovery regardless of whether they receive early, goal-directed mobilization. We examined the hypothesis that intensive care unit (ICU) patients presenting with moderate acuity of illness derive the greatest benefit from early, goal-directed mobilization.

In the SOMS trial [3], randomization was stratified based on the immediate Acute Physiology and Chronic Health Evaluation II (APACHE II) score. Patients received either standard of care or early, goal-directed mobilization. The primary endpoint, functional independence at hospital discharge, was defined as a minimal modified Functional Independence Measure score (mmFIM: range 0–8) of 8. Secondary outcome was speed of mobility progress (change in achieved SOMS level over time). Patients were classified into tertiles according to APACHE II score; low acuity as APACHE II ≤ 13 (1st tertile), moderate acuity as APACHE II 14–20 (2nd tertile) and high acuity as APACHE II ≥ 21 (3rd tertile) (Table 1). Multivariable logistic regression controlling for age and gender was used for binary outcomes and linear regression for continuous outcomes.

**Table 1:** Baseline characteristics of all patients divided by intervention and acuity of illness at ICU admission

	Control group			Intervention group			
	Low acuity of illness (APACHE II score ≤ 13)	Moderate acuity of illness (APACHE II score 14–20)	High acuity of illness (APACHE II score ≥ 21)	Low acuity of illness (APACHE II score ≤ 13)	Moderate acuity of illness (APACHE II score 14–20)	High acuity of illness (APACHE II score ≥ 21)	*p* for interaction
	*n* = 36	*n* = 29	*n* = 31	*n* = 38	*n* = 34	*n* = 32	
Age—median [IQR]	57 [34, 68]	64 [46, 77]	66 [56, 79]	52 [39, 67]	67 [51, 74]	67 [60, 75]	0.113
Female gender—*n* (%)	15 (42)	10 (34)	10 (32)	17 (45)	11 (32)	11 (34)	0.716
GCS—median [IQR]	10.0 [9.0, 11.5]	9.0 [8.0, 10.0]	9.0 [6.0, 10.0]	10.0 [9.0, 12.0]	9.0 [8.0, 10.0]	8.50 [5.5, 9.5]	0.927
APACHE II—median [IQR]	10.0 [7.0, 12.0]	17.0 [16.0, 19.0]	25.0 [22.0, 28.0]	10.5 [8.0, 12.0]	17.0 [15.0, 19.0]	26.0 [22.0, 29.0]	0.048
Charlson Comorbidity Index—mean ± SD	1.69 ± 2.20	3.16 ± 3.60	3.10 ± 2.85	2.55 ± 4.15	2.22 ± 2.60	3.79 ± 3.13	0.546
*Comorbidities*							
Myocardial infarction—*n* (%)	1 (3)	4 (14)	4 (13)	3 (8)	1 (3)	2 (6)	0.223
Cerebrovascular disease—*n* (%)	6 (17)	7 (24)	4 (13)	4 (11)	3 (9)	2 (6)	0.406
Diabetes mellitus—*n* (%)	2 (6)	8 (28)	7 (23)	3 (8)	4 (12)	9 (28)	0.457
Hemiplegia or paraplegia—*n* (%)	3 (8)	0 (0)	0 (0)	3 (8)	0 (0)	2 (6)	0.826
*Surgery classification*							
Abscess drainage—*n* (%)	5 (14)	0 (0)	0 (0)	3 (8)	3 (9)	0 (0)	0.723
Damage control surgery—*n* (%)	8 (22)	4 (14)	3 (10)	5 (13)	6 (18)	3 (9)	0.233
Aneurysm repair—*n* (%)	6 (17)	3 (10)	3 (10)	3 (8)	7 (20)	6 (19)	0.364
General surgery	2 (6)	3 (10)	10 (32)	7 (18)	5 (15)	8 (25)	0.096
Neurosurgery—*n* (%)	5 (14)	4 (14)	0 (0)	6 (16)	0 (0)	0 (0)	0.638
Other—*n* (%)	10 (27)	15 (51)	15 (48)	14 (37)	13 (38)	15 (46)	0.306

APACHE II, Acute Physiology and Chronic Health Evaluation II; IQR, interquartile range; GCS, Glasgow Coma Scale; SD, standard deviation; GI, gastrointestinal; *p* for interaction, *p* value for the interaction of the according study variable * intervention for the outcome functional independence at hospital discharge

Effectiveness of early, goal-directed mobilization was significantly modified by acuity of illness for the outcome functional independence at hospital discharge (*p* = 0.048 for the interaction “moderate acuity/non-moderate acuity[binary]*Intervention[binary]”). For patients with moderate acuity, predicted probability of functional independence was 44 per 100 patients who received early, goal-directed mobilization and 11 per 100 patients who did not (adjusted absolute risk difference [aARD] 33% [95% CI, 14 to 53%], *p* = 0.001). By contrast, in patients with low and high acuity, predicted probability of functional independence was 47 (low acuity) and 36 (high acuity) per 100 patients who received early, goal-directed mobilization and 34 (low acuity) and 30 (high acuity) per 100 patients who did not (aARD low acuity: 13% [95% CI, − 8 to 34%], *p* = 0.234; aARD high acuity: 6% [95% CI, − 17 to 29%], *p* = 0.632 [Fig. 1]).

**Fig. 1:**
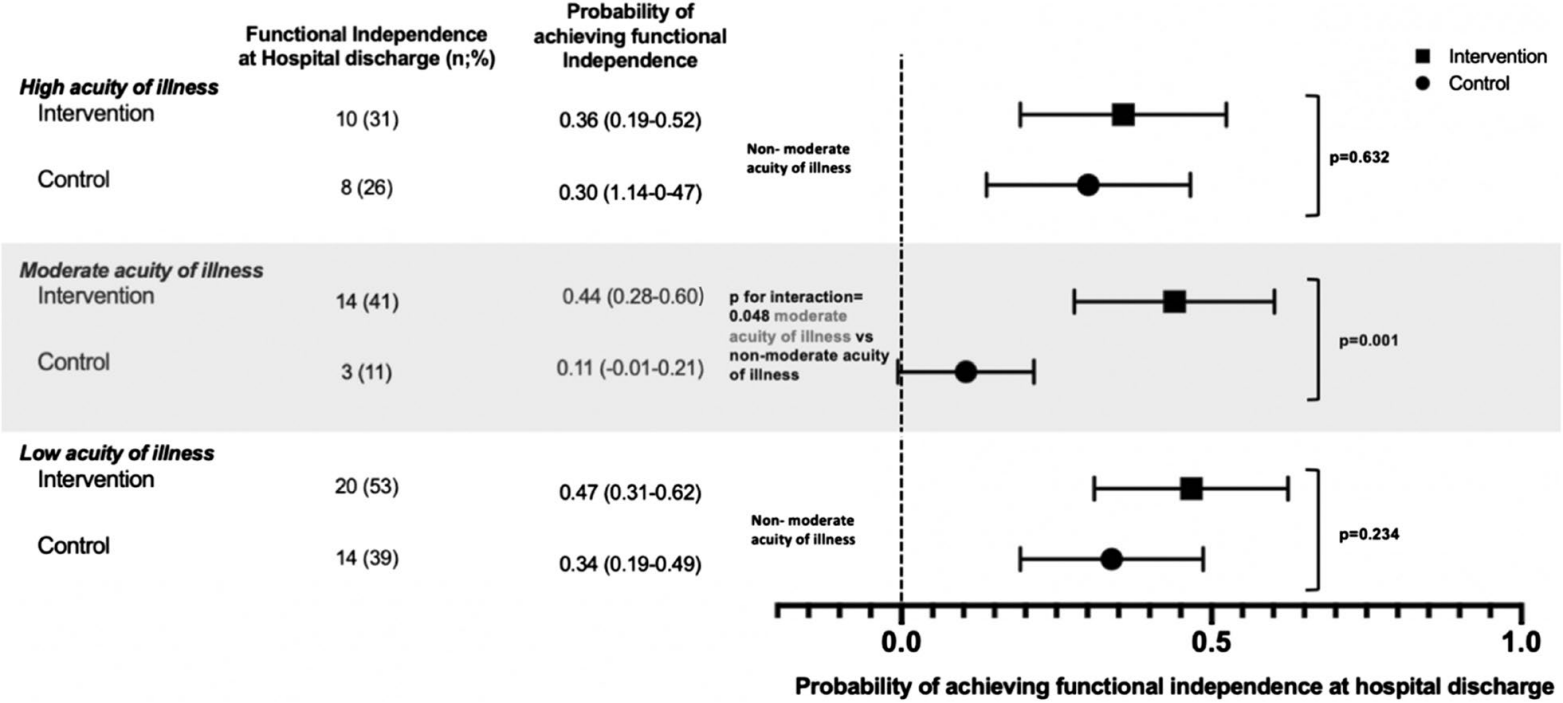
Predicted probability of achieving functional independence at hospital discharge by acuity of illness level. Predicted probabilities were calculated for early, goal-directed mobilization (intervention) and standard of care (control). The *x*-axis represents the predicted probability of achieving functional independence which can be evaluated for each acuity of illness level represented on the *y*-axis. Horizontal bars represent 95% confidence intervals. *p* values derived from subgroup analyses for the outcome functional independence comparing intervention versus control for each acuity level were adjusted for age and gender and are displayed on the right

Speed of mobility progress is an important outcome predictor [4]. We found that slope (speed of mobility recovery) was significantly higher in patients with moderate acuity who received early, goal-directed mobilization compared to patients who did not (*p* = 0.018). By contrast, among patients with low and high acuity, speed of mobility progress did not differ significantly between treatment groups (*p* = 0.30 and *p* = 0.18, respectively). The beneficial effect of early, goal-directed mobilization on speed of mobility progress in patients with moderate acuity may contribute to the improved functional outcomes observed.

Only two randomized controlled trials examining the effectiveness of early, goal-directed mobilization on functional outcomes provide APACHE II scores [1]. Schweickert et al. enrolled patients with moderate acuity (median APACHE II 19–20) and demonstrated that early mobilization improved functional outcomes; by contrast, Kayambu et al. did not observe beneficial effects of early mobilization on functional outcomes in patients with higher acuity (mean APACHE II 27–28 [1]). Impaired cardiorespiratory reserve and decreased capacity for anabolism in patients with high acuity may also limit effectiveness of early mobilization [2, 5, 6].

In our cohort, patients with moderate acuity in the control group carried an underrecognized need for mobilization therapy. They received the lowest number of physiotherapist visits (14% of ICU days with physiotherapist visits vs. 25% and 20% for high and low acuity, respectively), and had the lowest likelihood of achieving functional independence.

Early, goal-directed mobilization is a resource intensive intervention that cannot be applied to all ICU patients. Our data support the view that patients with low acuity are in less need of early, goal-directed mobilization. Focusing time and effort on patients benefitting most is probably more cost-effective.

## Data Availability

Questions about data are handled by the corresponding author.

## Code availability

Questions about code availability are handled by the corresponding author.
